# Glycogen supercompensation in skeletal muscle after cycling or running followed by a high carbohydrate intake the following days: a systematic review and meta-analysis

**DOI:** 10.3389/fphys.2025.1620943

**Published:** 2025-08-18

**Authors:** Kristian Solem, Matthieu Clauss, Jørgen Jensen

**Affiliations:** Department of Physical Performance, Norwegian School of Sport Sciences, Oslo, Norway

**Keywords:** muscle glycogen supercompensation, protein expression, glycogen synthase, signaling, phosphorylation, systematic review, meta-analysis, meta-regression

## Abstract

**Introduction:**

Bergström and Hultman demonstrated that exhaustive exercise depleting muscle glycogen followed by three days on a carbohydrate-rich diet resulted in a doubling of the glycogen content. Although many studies have confirmed this finding, the magnitude of glycogen supercompensation and the mechanisms behind elevated glycogen content after exercise remain unclear. This systematic review meta-analyzed investigations on muscle glycogen supercompensation after exercise and 3–5 days on a high-carbohydrate diet. Meta-regression analyses were conducted to explore the influence of specific variables on muscle glycogen supercompensation.

**Methods:**

A systematic search was performed for published studies in PubMed and Web of Science in March 2025. Inclusion criteria were: 1) reported basal glycogen values after a mixed diet; 2) included an exercise session prior to the dietary intervention; 3) utilized high carbohydrate intake after exercise to supercompensate glycogen stores; 4) measured muscle glycogen content after 3–5 days on a high-carbohydrate diet; and 5) reported quantitative data on glycogen. Data were extracted to compare muscle glycogen supercompensation following cycling and running exercises, followed by a 3–5-day high-carbohydrate diet. Meta-analyses were performed using the mean difference (MD) with 95% confidence intervals (CI).

**Results:**

A total of 30 studies published between 1966 and 2020 were included, comprising 319 participants (271 males and 48 females). Glycogen increased by 269.7 ± 29.2 mmol⋅kg^−1^ dry weight (dw) (95%CI [212.4, 327.0]; p < 0.001) after cycling exercise and by 156.5 ± 48.6 mmol⋅kg^−1^ dw (95%CI [61.3, 251.7]; p = 0.001) after running exercise. Muscle glycogen supercompensation after cycling was positively associated with percent carbohydrate in the diet (p < 0.001) and negatively associated with basal glycogen concentration (p = 0.011) and glycogen concentration after exercise (p < 0.001).

**Conclusion:**

Muscle glycogen supercompensation occurs following both cycling and running after 3–5 days on a high-carbohydrate diet, with a greater magnitude observed after cycling compared to running. The magnitude of glycogen supercompensation after cycling is influenced by basal glycogen levels, glycogen content after exercise, and the relative carbohydrate content of the diet.

## 1 Introduction

In 1966, Bergström and Hultman described that depleting the glycogen stores in skeletal muscle followed by 3 days on a carbohydrate-rich diet doubled the glycogen content (Bergström and Hultman, 1966). They confirmed the glycogen supercompensation in several studies the following year (Ahlborg et al., 1967; Bergström et al., 1967; Hultman and Bergström, 1967). More important, they showed that muscle glycogen was a determining factor for exercise performance, which spurred the interest in glycogen supercompensation further (Bergström et al., 1967).

The mechanisms for muscle glycogen supercompensation remain unknown, but several contributing factors have been enlightened. It seems clear that glycogen depletion is required. The strongest evidence that glycogen depletion is necessary is that Bergström and Hultman (1966) used one-legged cycling and reported only glycogen supercompensation in the exercise leg whereas no supercompensation occurred in the rested leg. In rats, muscle glycogen also doubles when refed after 24 h fasting, which reduces glycogen content by ∼50% (Jensen et al., 1997; 2006; Kolnes et al., 2015). High intake of carbohydrate is also necessary, and prolonged endurance training increases glycogen content in muscles (Ivy, 2000; Betts and Williams, 2010; Jensen et al., 2011).

Many studies have reported glycogen supercompensation after exercise followed by intake of a carbohydrate-rich diet for several days without further exercise (Bergström and Hultman, 1966; Ahlborg et al., 1967; Bergström et al., 1967; Gollnick et al., 1972; Kochan et al., 1979; Shiose et al., 2016). These studies have used different exercise and diet protocols, and it is not surprising that the magnitude of glycogen supercompensation differs. Moreover, glycogen depletion after running seems lower, and lack of glycogen supercompensation after running has been reported (Blom et al., 1987; Madsen et al., 1990). Recently, Jensen et al. (2020) repeated the study by Bergström et al. (1967) but did not find significant glycogen supercompensation. Other studies have also found little glycogen supercompensation after exercise followed by 3–5 days on a carbohydrate-rich diet (Roedde et al., 1986; Lamb et al., 1991; Tarnopolsky et al., 1995).

The aim of the present study was to conduct a systematic review and meta-analysis to quantify the size of glycogen supercompensation after an exercise session followed by 3–5 days with high intake of carbohydrate. The analyses were conducted separately for cycling and running because of the different mechanical challenges and degrees of glycogen depletion. Finally, meta-regressions were performed to explore potential factors influencing the magnitude of glycogen supercompensation in cycling studies.

## 2 Materials and methods

### 2.1 Search strategy and selection of studies

The current systematic review and meta-analysis were carried out following the guidelines of the Preferred Reporting Items for Systematic Review and Meta-Analyses (PRISMA) (Page et al., 2021). The review protocol was not prospectively registered with PROSPERO or any other registry. The search for published studies on the topic was conducted in the databases PubMed and Web of Science in March 2025. Search terms included the following syntax: “muscle glycogen” AND (“supercompensation” OR “Hultman” OR “carbohydrate loading”) AND “human” NOT (“brain” OR “liver”). The search was conducted without any restriction for the year of publication, and the language filter was used to select only studies written in English. The complete search strings used for each database is available in Supplementary Table S1. All titles retrieved from the search were cross-referenced to identify duplicates. Titles and abstracts were then screened to determine eligibility for full-text review. Secondary searches consisted of screening the reference lists of the included studies. The search for published studies was performed by one author (K.S.), while all potentially relevant studies were screened and evaluated for inclusion in the analysis by all authors (K.S., M.C., and J.J.).

For the articles obtained through the search, the following inclusion criteria were applied to select studies: 1) reported basal glycogen values after a mixed diet; 2) included an exercise session before the dietary intervention started; 3) utilized a high-carbohydrate intake after exercise to supercompensate glycogen stores; 4) measured muscle glycogen content after 3–5 days on a high-carbohydrate diet; and 5) measured muscle glycogen content using a biochemical assay or nuclear magnetic resonance (NMR) spectroscopy. Only studies utilizing cycling or running were included.

### 2.2 Data extraction

Information about participants, interventions, comparisons, outcomes, and study design (PICOS) was collected following the PRISMA methodology (Page et al., 2021). The extracted data included: 1) the number and characteristics of participants; 2) skeletal muscle glycogen concentration measured before exercise, after exercise, and after a 3–5-day high-carbohydrate diet; 3) dietary intake before and after glycogen depletion, including energy intake (kcal⋅kg^-1^⋅day^-1^), macronutrient composition (percentage of total energy intake), and carbohydrate intake (g⋅kg^-1^⋅day^-1^); and 4) details of the glycogen depletion protocol, including the type, duration, and intensity of exercise. Glycogen breakdown during exercise was calculated as the absolute difference between basal glycogen content and the glycogen content immediately after exercise. In instances where data were presented in a graphical format, images were enlarged to improve the precision of the data estimates, and values were extracted using WebPlotDigitizer (PlotDigitizer, Version 3.1.5, 2023). Two authors (K.S. and M.C.) independently extracted the data and compared the results to ensure accuracy and consistency. Any disagreement was resolved through discussion, and a third author (J.J.) was involved in the process.

#### 2.2.1 Extraction of multiple study groups from included studies

In studies with multiple groups under identical experimental conditions, data were aggregated and averaged for use in the present review. Alternatively, when studies included several independent experimental conditions and provided data for each of them, data from each condition were used separately. In the meta-analysis for cycling, subgroup-level data extraction was possible in seven studies based on differences in pre-intervention diet (Ahlborg et al., 1967; Bergström et al., 1967; Hultman and Bergström, 1967), training status between groups (Roedde et al., 1986), or sex (Tarnopolsky et al., 1995; 2001; James et al., 2001). Similarly, in the meta-analysis for running, data extraction from multiple groups was possible in five studies, based on differences in pre-intervention diet (Sherman et al., 1981), depletion protocols (Roberts et al., 1988; Fogelholm et al., 1991), training status (Blom et al., 1987), or carbohydrate supplementation (Lamb et al., 1991).

#### 2.2.2 Glycogen unit conversion

To standardize reported values of muscle glycogen concentration, unit conversions were applied when data were presented in formats other than mmol⋅kg^-1^ dry weight (dw). Values reported as g⋅100 g^-1^ wet weight (ww) or g⋅kg^-1^ ww were first converted to mmol⋅kg^-1^ ww using a glycogen molecular weight of 162 g⋅mol^-1^ (empirical formula for glycogen of (C_6_H_10_O_5_)_n_). In studies where glycogen was measured with NMR, concentrations were reported as mmol⋅L^-1^ and converted to mmol⋅kg^-1^ ww by assuming a muscle density of 1.06 kg⋅L^-1^ (Mendez and Keys, 1960). All values converted to, or originally expressed as, mmol⋅kg^-1^ ww were then converted to mmol⋅kg^-1^ dw using a fixed muscle water content of 76% (Karlsson et al., 1974; Blomstrand and Saltin, 1999). Although muscle water content has been reported to increase to 77%–78% during exercise (Karlsson et al., 1974), no significant differences were observed between pre- and post-exercise (Blomstrand and Saltin, 1999). Therefore, a constant value of 76% was used for all conversions. Additionally, values reported as mmol⋅kg^-1^ protein were converted to mmol⋅kg^-1^ dw by assuming that protein comprises 64% of dry muscle mass.

### 2.3 Risk of bias assessment

Small-study effects and potential publication bias were assessed using Egger’s regression test (Egger et al., 1997) and visualized with contour-enhanced funnel plots (Peters et al., 2008). Egger’s test was used to statistically evaluate funnel plot asymmetry, which may suggest the presence of publication bias. All authors independently examined the funnel plots, and any discrepancies in interpretation were resolved through discussion.

### 2.4 Statistical analyses

All statistical analyses and the creation of figures were performed using R (Version 4.3.2, R Core Team, Vienna, Austria, 2023). Statistical significance was set at *p* < 0.05. Data in text and tables are presented as mean ± standard error of the mean (SEM). In forest plots, effect sizes are reported as mean difference (MD) with 95% confidence intervals (CI), and in meta-regression figures, average regression lines with 95% CIs are shown.

Separate meta-analyses were conducted to examine the effects of cycling and running, followed by a 3–5-day high-carbohydrate diet, on muscle glycogen supercompensation. Cycling and running were analyzed separately because running imposes greater mechanical strain on the muscles than cycling, which may influence glycogen supercompensation (Costill et al., 1990). Meta-regression analyses were performed to investigate the influence of continuous moderator variables on the primary outcome in studies using cycling as the exercise modality. The moderator variables included: (1) carbohydrate intake relative to body mass (g·kg^-1^·day^-1^); (2) carbohydrate intake as a percentage of total energy intake (%); (3) basal glycogen concentration (mmol·kg^-1^ dw); (4) glycogen concentration immediately after exercise (mmol⋅kg^-1^ dw); (5) glycogen breakdown during exercise (mmol·kg^-1^ dw); (6) glycogen concentration during supercompensation (mmol·kg^-1^ dw); and (7) maximal oxygen uptake (VO_2max_; ml⋅kg^-1^⋅min^-1^). The number of running studies was limited, and reporting of moderator variables was often insufficient (Karlsson and Saltin, 1971; Sherman et al., 1981; Roberts et al., 1988; Fogelholm et al., 1991; Lamb et al., 1991; Price et al., 2000; 2003). We judged that the available data were inadequate for conducting meaningful meta-regression analyses for running.

Effect sizes and their corresponding sampling variances were computed using the ‘escalc’ function from the ‘metafor’ package (Viechtbauer, 2010) in R, with MD selected as the effect size metric given the common reporting of glycogen concentration in mmol⋅kg^-1^ dw. Meta-analyses and meta-regressions were conducted using random-effects models fitted with the ‘rma’ function, with the restricted maximum likelihood method used to estimate the between-study variance (τ^2^). Forest plots were created using the ‘forest’ function from ‘metafor’, and meta-regression results were visualized using the ‘regplot’ function in R.

A standardized measure of homogeneity (I^2^ statistic) was calculated to evaluate the level of heterogeneity in the included studies. I^2^ values between 25% and 50% were interpreted as indicating a low level of heterogeneity, values between 50% and 75% as moderate, and values >75% as high heterogeneity (Higgins and Thompson, 2002).

## 3 Results

### 3.1 Study selection

**FIGURE 1:**
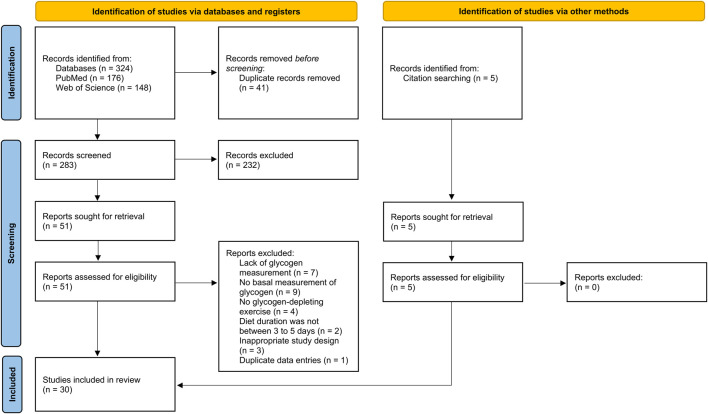
PRISMA 2020 flow diagram illustrating the selection process for studies identified through database and other source searches in the current systematic review.

A total of 324 articles were identified in the initial database searches, and an additional five articles were found by screening the reference lists of relevant papers. After removing duplicates, 288 studies remained for title and abstract screening. Based on this screening, 232 studies were excluded for one or more of the following reasons: they were reviews or meta-analyses; included participants with metabolic conditions; included animals as study samples; or were unrelated to the topic of this review. The remaining 56 full-text articles were assessed for eligibility according to the predefined inclusion criteria. Of these, 26 studies were excluded for the following reasons: (a) lack of glycogen measurement (n = 7); (b) no basal measurement of glycogen (n = 9); (c) no glycogen-depleting exercise (n = 4); (d) diet duration was not between 3 and 5 days (n = 2); (e) inappropriate study design (n = 3); or (f) duplicate data entries (n = 1). Following this process, 30 studies published between 1966 and 2020 were included in the final analyses. A summary of this selection process is presented in Figure 1.

### 3.2 Meta-analyses

#### 3.2.1 Cycling

**FIGURE 2:**
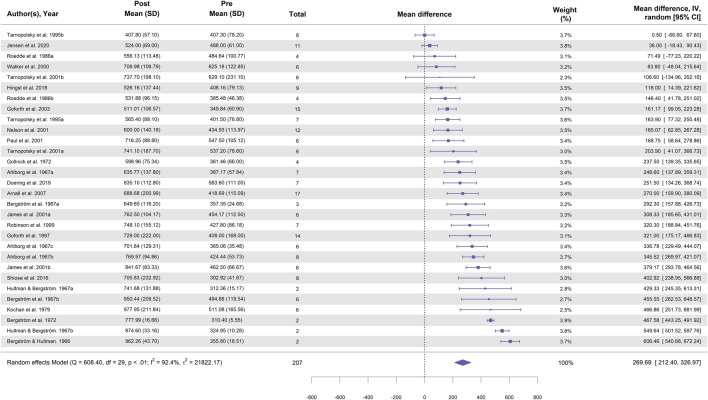
Forest plot showing the effect of cycling followed by a 3–5-day high-carbohydrate diet on muscle glycogen supercompensation. Effect sizes are expressed as mean difference (MD) in glycogen concentration (mmol⋅kg^-1^ dw) with 95% confidence intervals (CI) from 22 studies (30 study groups). The diamond represents the pooled effect size (95% CI) calculated using a random-effects model. The size of each square indicates the weight of the corresponding study group in the analysis.

In total, 30 study groups from 22 studies were included in the meta-analysis for cycling (Table 1). Figure 2 illustrates the effect of cycling followed by a high-carbohydrate diet on glycogen supercompensation, showing a statistically significant increase in muscle glycogen concentration, with a MD of 269.7 ± 29.2 mmol⋅kg^-1^ dw (95% CI [212.4, 327.0]; *p* < 0.001; I^2^ = 92.4%).

**TABLE 1:** Overview of study groups investigating muscle glycogen supercompensation following cycling and a subsequent high-carbohydrate diet.

Study	Participant characteristics (number, VO_2_max/VO_2_peak; ml·kg^-1^·min^-1^)	Diet before exercise	Type of exercise	Duration	Intensity	Exercise during loading phase	Diet after exercise	CHO intake (g·kg^1^·day^-1^)	Days	Glycogen concentration (mmol·kg^-1^dw)
Bergström and Hultman (1966)	n = 2♂		One-legged intervals until exhaustion (bouts of 5–20 min)		1,200 kpm⋅min^-1^		∼34 kcal·kg^-1^ CHO: 90%	∼7.8	3	Baseline: 356 ± 13 Post: 962 ± 31 After exercise: 29 ± 4
Ahlborg et al. (1967)a	n = 7♂		Continuous until exhaustion	72 min	85% of W_170_		45 kcal·kg^-1^ CHO: 90% FAT: 5% PRO: 5%	10.2	3	Baseline: 387 ± 22 Post: 636 ± 52 After exercise: 75 ± 19
Ahlborg et al. (1967)b	n = 8♂	42 kcal·kg^-1^ CHO: 0% FAT: 65% PRO: 35%	Continuous until exhaustion	66 min	85% of W_170_		42 kcal·kg^-1^ CHO: 90% FAT: 5% PRO: 5%	9.3	3	Baseline: 424 ± 19 Post: 770 ± 34 After exercise: 123 ± 23
Ahlborg et al. (1967)c	n = 6♂	45 kcal·kg^-1^ CHO: 0% FAT: 65% PRO: 35%	Continuous until exhaustion	64 min	85% of W_170_		45 kcal·kg^-1^ CHO: 90% FAT: 5% PRO: 5%	10.1	3	Baseline: 365 ± 14 Post: 702 ± 53 After exercise: 77 ± 16
Bergström et al. (1967)a	61.3 ± 2.2 n = 3♂		Intervals until exhaustion (bouts of 60, 45, 30, 30, and 30 min)	89 ± 3 min	75% of VO_2max_		41 kcal·kg^-1^ CHO: 82% FAT: 0% PRO: 18%	8.4	3	Baseline: 357 ± 14 Post: 650 ± 67 After exercise: 26 ± 9
Bergström et al. (1967)b	59.2 ± 2.6 n = 6♂	40 kcal·kg^-1^ CHO: 0% FAT: 46% PRO: 54%	Intervals until exhaustion (bouts of 60, 45, 30, 30 and 30 min)	59 ± 6 min	75% of VO_2max_		40 kcal·kg^-1^ CHO: 82% FAT: 0% PRO: 18%	8.2	3	Baseline: 495 ± 49 Post: 950 ± 86 After exercise: 49 ± 9
Hultman and Bergström (1967)a	n = 2♂	CHO: 5%	One-legged continuous cycling until exhaustion		60% of W_170_		CHO: 95%		3	Baseline: 312 ± 11 Post: 742 ± 93 After exercise: 34 ± 15
Hultman and Bergström (1967)b	n = 2♂	0 kcal·kg^-1^ CHO: 0% FAT: 0% PRO: 0%	One-legged continuous cycling until exhaustion		60% of W_170_		CHO: 95%		4	Baseline: 325 ± 7 Post: 875 ± 23 After exercise: 38 ± 21
Bergström et al. (1972)	n = 2♂		One-legged intervals until exhaustion (bouts of 5–20 min)				CHO: 95%		3	Baseline: 310 ± 4 Post: 778 ± 12 After exercise: 0 ± 0
Gollnick et al. (1972)	57.0 ± 2.6 n = 4♂	47 kcal·kg^-1^ CHO: 3% FAT: 51% PRO: 46%	Continuous	30 min	74% of VO_2peak_		47 kcal·kg^-1^ CHO: 69%	8.0	3	Baseline: 361 ± 33 Post: 599 ± 38 After exercise: 27 ± 4
Kochan et al. (1979)	n = 6♂	50 kcal·kg^-1^ CHO: 10% FAT: 57% PRO: 33%	One-legged intervals (bouts of 15 min)	60 min	75% of VO_2max_		50 kcal·kg^-1^ CHO: 90% FAT: 3% PRO: 7%	11.3	4	Baseline: 511 ± 68 Post: 978 ± 86 After exercise: 53 ± 9
Roedde et al. (1986)a	63.0 ± 2.5 n = 4♂		Continuous until exhaustion followed by five 30-s sprints		∼73% of VO_2max_		CHO: 68%		5	Baseline: 485 ± 50 Post: 556 ± 57 After exercise: 94 ± 18
Roedde et al. (1986)b	48.0 ± 3.0 n = 4♂		Continuous until exhaustion followed by five 30-s sprints		∼73% of VO_2max_		CHO: 68%		5	Baseline: 385 ± 23 Post: 532 ± 48 After exercise: 78 ± 33
Tarnopolsky et al. (1995)a	64.6 ± 1.2 n = 7♂	45 kcal·kg^-1^ CHO: 58% FAT: 30% PRO: 12%	Two bouts of continuous cycling separated by a ∼20 min break	First bout: 1 h Second bout: exhaustion	75% of VO_2peak_ 85% of VO_2peak_		45 kcal·kg^-1^ CHO: 73% FAT: 14% PRO: 13%	8.2	4	Baseline: 402 ± 29 Post: 565 ± 33 After exercise: 107 ± 10
Tarnopolsky et al. (1995)b	53.4 ± 2.1 n = 8♀	33 kcal·kg^-1^ CHO: 57% FAT: 31% PRO: 12%	Two bouts of continuous cycling separated by a ∼20 min break	First bout: 1 h Second bout: exhaustion	75% of VO_2peak_ 85% of VO_2peak_		34 kcal·kg^-1^ CHO: 75% FAT: 15% PRO: 10%	8.6	4	Baseline: 407 ± 28 Post: 408 ± 20 After exercise: 79 ± 15
Goforth et al. (1997)	54.0 ± 1.8 n = 14♂	28 kcal·kg^-1^ CHO: 10% FAT: 47% PRO: 44%	Continuous followed by three 1-min sprints	Day 1: 115 min Day 2: 40 min Day 3: 40 min	75% of VO_2peak_		43 kcal·kg^-1^ CHO: 85% FAT: 7% PRO: 8%	9.2	4	Baseline: 408 ± 45 Post: 729 ± 59
Robinson et al. (1999)	n = 7♂		Continuous until near exhaustion followed by intervals (∼5 min) at same intensity		W_165_		CHO: 80%		5	Baseline: 428 ± 33 Post: 748 ± 59 After exercise: 30 ± 12
Walker et al. (2000)	56.4 ± 1.5 n = 6♀	39 kcal·kg^-1^ CHO: 48% FAT: 34% PRO: 19%	Continuous until exhaustion	Day 1: 90 min Day 2 and 3: 45 min	80% of VO_2peak_	Day 4 and 5: 20 min	39 kcal·kg^-1^ CHO: 78% FAT: 10% PRO: 11%	8.2	4	Baseline: 625 ± 50 Post: 709 ± 45
James et al. (2001)a	n = 6♂	48 kcal·kg^-1^ CHO: 51%	Continuous until exhaustion	98 ± 10 min	∼64% of VO_2peak_		51 kcal·kg^-1^ CHO: 82%	10.5	3	Baseline: 454 ± 46 Post: 762 ± 43
James et al. (2001)b	n = 6♀	41 kcal·kg^-1^ CHO: 51%	Continuous until exhaustion	92 ± 5 min	∼61% of VO_2peak_		47 kcal·kg^-1^ CHO: 82%	9.9	3	Baseline: 462 ± 27 Post: 842 ± 34
Nelson et al. (2001)	n = 12♂		One-legged continuous until exhaustion followed by knee-extensions		75% of PPO		CHO: 80% FAT: 10% PRO: 10%	6.6	3	Baseline: 435 ± 33 Post: 600 ± 40
Paul et al. (2001)	49 n = 6♀	39 kcal·kg^-1^ CHO: 58% FAT: 23% PRO: 19%	Intervals (6 x (12 + 1 + 2 min))	90 min	72% of VO_2max_(12 min) 100% of VO_2max_(1 min) 50% of VO_2max_(2 min)	Day 1 and 2: 30 min	45 kcal·kg^-1^ CHO: 80% FAT: 11% PRO: 9%	9.0	3	Baseline: 548 ± 43 Post: 716 ± 36
Tarnopolsky et al. (2001)a	62.8 ± 2.2 n = 6♂	41 kcal·kg^-1^ CHO: 58% FAT: 28% PRO: 15%	Continuous followed by intervals	60 min + 5 × 2 min	65% of VO_2peak_ 85% of VO_2peak_	Day 1: 60 min Day 2: 45 min Day 3: 30 min	55 kcal·kg^-1^ CHO: 75% FAT: 14% PRO: 11%	10.5	5	Baseline: 537 ± 32 Post: 741 ± 77
Tarnopolsky et al. (2001)b	53.3 ± 2.6 n = 6♀	34 kcal·kg^-1^ CHO: 59% FAT: 26% PRO: 14%	Continuous followed by intervals	60 min + 5 × 2 min	65% of VO_2peak_ 85% of VO_2peak_	Day 1: 60 min Day 2: 45 min Day 3: 30 min	46 kcal·kg^-1^ CHO: 75% FAT: 15% PRO: 10%	8.8	5	Baseline: 629 ± 94 Post: 738 ± 81
Goforth et al. (2003)	49.1 ± 1.4 n = 15♂	44 kcal·kg^-1^ CHO: 44% FAT: 38% PRO: 18%	Continuous followed by sprints until exhaustion	120 min + 5–21 sprints	65% of VO_2peak_ 120% of VO_2peak_	Day 1 and 2: 20 min	46 kcal·kg^-1^ CHO: 80% FAT: 10% PRO: 10%	9.2	3	Baseline: 350 ± 16 Post: 511 ± 28 After exercise: 149 ± 24
Arnall et al. (2007)	57.5 ± 1.5 n = 17♂		Continuous until exhaustion		70%–75% of HR_max_		43 kcal·kg^-1^ CHO: 85% FAT: 7% PRO: 8%	9.1	3	Baseline: 419 ± 28 Post: 689 ± 49
Shiose et al. (2016)	44.8 ± 2.4 n = 8♂	32 kcal·kg^-1^ CHO: 58% FAT: 28% PRO: 14%	Continuous + Intervals + Continuous until exhaustion	60 min + 5 × 1 min	70% of VO_2max_+ 100% of VO_2max_+ 100% of VO_2max_		57 kcal·kg^-1^ CHO: 84% FAT: 8% PRO: 8%	12.4	3	Baseline: 303 ± 15 Post: 706 ± 82 After exercise: 107 ± 13
Hingst et al. (2018)	45.0 ± 1.3 n = 9♂		One-legged continuous knee-extensions followed by intervals until exhaustion	60 min	84% of PWL 100, 90, 80, 70, and 60% of PWL		CHO: 80% FAT: 10% PRO: 10%		4	Baseline: 408 ± 26 Post: 526 ± 46 After exercise: 137 ± 17
Doering et al. (2019)	56.8 ± 1.4 n = 6♂/1♀	33 kcal·kg^-1^ CHO: 63%	Intervals until exhaustion (bouts of 2 min)		90, 80, 70, and 60% of PPO		59 kcal·kg^-1^ CHO: 71% FAT: 15% PRO: 11%	10.6	4	Baseline: 584 ± 42 Post: 835 ± 43
Jensen et al. (2020)	59.4 ± 2.2 n = 11♂	40 kcal·kg^-1^ CHO: 5% FAT: 49% PRO: 47%	Continuous (one bout of 60 min + bouts of 30 min thereafter)		75% of VO_2max_		42 kcal·kg^-1^ CHO: 78% FAT: 7% PRO: 14%	8.0	3	Baseline: 488 ± 18 Post: 524 ± 21 After exercise: 178 ± 17

CHO, carbohydrate; FAT, fat; PRO, protein; ♂, male; ♀, female; W, watt; W170, watt at a heart rate of 170 beats•min^-1^; W165, watt at a heart rate of 165 beats•min^-1^; VO_2_max, maximal oxygen uptake; VO_2_peak, highest oxygen uptake measured; HRmax, maximal heart rate; RPM, revolutions per minute; PWL, peak workload; PPO, peak power output.

#### 3.2.2 Running

**FIGURE 3:**
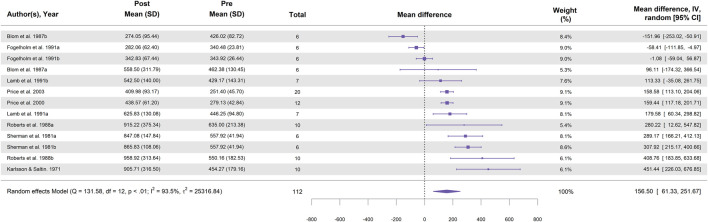
Forest plot showing the effect of running followed by a 3–5-day high-carbohydrate diet on muscle glycogen supercompensation. Effect sizes are expressed as mean difference (MD) in glycogen concentration (mmol⋅kg^-1^ dw) with 95% confidence intervals (CI) from eight studies (13 study groups). The diamond represents the pooled effect size (95% CI) calculated using a random-effects model. The size of each square indicates the weight of the corresponding study group in the analysis.

In total, 13 study groups from eight studies were included in the meta-analysis for running (Table 2). Figure 3 illustrates the effect of running followed by a high-carbohydrate diet on muscle glycogen supercompensation, showing a statistically significant increase in muscle glycogen concentration, with a MD of 156.5 ± 48.6 mmol⋅kg^-1^ dw (95% CI [61.3, 251.7]; *p* = 0.001; I^2^ = 93.5%).

**TABLE 2:** Overview of study groups investigating muscle glycogen supercompensation following running and a subsequent high-carbohydrate diet.

Study	Participant characteristics (number, VO_2_max/VO_2_peak; ml·kg^-1^·min^-1^)	Diet before exercise	Type of exercise	Duration	Intensity	Exercise during loading phase	Diet after exercise	CHO intake (g·kg^-1^·day^-1^)	Days	Glycogen concentration (mmol·kg^-1^dw)
Karlsson and Saltin (1971)	67.7 ± 2.8 n = 10		Continuous	Days 1–3: <2 h	Intense			9.0	3	Baseline: 454 ± 57 Post: 906 ± 100
Sherman et al. (1981)a	65.3 ± 3.4 n = 6♂	40 kcal·kg^-1^ CHO: 50% FAT: 32% PRO: 18%	Continuous	Day 1: 90 min Days 2 and 3: 40 min Day 4: 20 min	73% of VO_2max_	Day 5: 20 min	44 kcal·kg^-1^ CHO: 70% FAT: 20% PRO: 12%	7.8	3	Baseline: 558 ± 17 Post: 847 ± 60
Sherman et al. (1981)b	65.3 ± 3.4 n = 6♂	40 kcal·kg^-1^ CHO: 15% FAT: 66% PRO: 22%	Continuous	Day 1: 90 min Days 2 and 3: 40 min Day 4: 20 min	73% of VO_2max_	Day 5: 20 min	44 kcal·kg^-1^ CHO: 70% FAT: 20% PRO: 12%	7.8	3	Baseline: 558 ± 17 Post: 866 ± 44
Blom et al. (1987)a	69.0 ± 1.0 n = 6♂	45 kcal·kg^-1^ CHO: 53%	Intervals until exhaustion (bouts of 30, 25, 20, 15, 10, and 5 min)	114 ± 5 min	79% of VO_2max_		52 kcal·kg^-1^ CHO: 69%	9.0	3	Baseline: 462 ± 53 Post: 559 ± 127 After exercise: 236 ± 60
Blom et al. (1987)b	51.0 ± 4.0 n = 6♂	40 kcal·kg^-1^ CHO: 53%	Intervals until exhaustion (bouts of 30, 25, 20, 15, 10, and 5 min)	90 ± 6 min	73% of VO_2max_		46 kcal·kg^-1^ CHO: 69%	7.9	3	Baseline: 426 ± 34 Post: 274 ± 39 After exercise: 107 ± 19
Roberts et al. (1988)a	n = 10♂	CHO: 15%	Continuous	Day 1: ∼2 h Days 2 and 3: ∼50 min Day 4: ∼35 min Day 6: ∼20 min			CHO: 70%		3	Baseline: 635 ± 67 Post: 915 ± 119
Roberts et al. (1988)b	n = 10♂	CHO: 50%	Continuous	Day 1: ∼2 h Days 2 and 3: ∼50 min Day 4: ∼35 min Day 6: ∼20 min			CHO: 70%		3	Baseline: 550 ± 58 Post: 959 ± 99
Fogelholm et al. (1991)a	n = 6♂	33 kcal·kg^-1^ CHO: 37%	Continuous followed by sprints until exhaustion	Day 1: 74 min +15 × 200-m sprints (total 95–100 min) Day 2: 80–90 min	70%–80% of VO_2max_	Days 3–6: 45–60 min	52 kcal·kg^-1^ CHO: 70%	9.1	5	Baseline: 341 ± 10 Post: 282 ± 25
Fogelholm et al. (1991)b	n = 6♂	29 kcal·kg^-1^ CHO: 31%	Continuous	Day 1: 95–100 min Day 2: 80–90 min	70%–80% of VO_2max_	Days 3–6: 45–60 min	57 kcal·kg^-1^ CHO: 67%	9.5	5	Baseline: 344 ± 11 Post: 343 ± 28
Lamb et al. (1991)a	64.5 ± 2.4 n = 7♂	52 kcal·kg^-1^ CHO: 20% FAT: 57% PRO: 23%	Continuous	Days 1 and 2: 40–80 min Day 3: ∼2 h	65%–75% of VO_2max_		52 kcal·kg^-1^ CHO: 90% FAT: 3% PRO: 8%	11.7	3.5	Baseline: 446 ± 36 Post: 626 ± 49
Lamb et al. (1991)b	65.9 ± 3.0 n = 7♂	50 kcal·kg^-1^ CHO: 20% FAT: 57% PRO: 23%	Continuous	Days 1 and 2: 40–80 min Day 3: ∼2 h	65%–75% of VO_2max_		50 kcal·kg^-1^ CHO: 90% FAT: 3% PRO: 8%	11.2	3.5	Baseline: 429 ± 54 Post: 543 ± 53
Price et al. (2000)	n = 5♂/7♀	45 kcal·kg^-1^ CHO: 20% FAT: 60% PRO: 20%	Continuous followed by single-leg toe raises	1 h + 5 min of toe raises for each leg	87% of HR_max_ ∼50% of MVC		45 kcal·kg^-1^ CHO: 90% FAT: 2% PRO: 8%	10.1	4	Baseline: 279 ± 12 Post: 439 ± 18
Price et al. (2003)	n = 12♂/8♀	45 kcal·kg^-1^ CHO: 20% FAT: 60% PRO: 20%	Continuous followed by single-leg toe raises	1 h + 5 min of toe raises for each leg	87% of HR_max_ ∼50% of MVC		45 kcal·kg^-1^ CHO: 90% FAT: 2% PRO: 8%	10.1	4	Baseline: 251 ± 10 Post: 410 ± 21 After exercise: 97 ± 2

CHO, carbohydrate; FAT, fat; PRO, protein; ♂, male; ♀, female; VO_2_max, maximal oxygen uptake; VO_2_peak, highest oxygen uptake measured; HRmax, maximal heart rate; MVC, maximum voluntary contraction.

#### 3.2.3 Role of gender

In total, 24 study groups from 19 studies included in the meta-analysis on cycling reported data on males only (n = 168). These results showed a statistically significant increase in muscle glycogen concentration among males, with a MD of 294.3 ± 32.0 mmol⋅kg^-1^ dw (95% CI [231.5, 357.1]; *p* < 0.001; I^2^ = 92.5%; Supplementary Figure S1). For females, data were available from five study groups across five studies (n = 32). The results showed a statistically significant increase in muscle glycogen concentration among females, with a MD of 151.6 ± 70.9 mmol⋅kg^-1^ dw (95% CI [12.8, 290.5]; *p* = 0.032; I^2^ = 87.9%; Supplementary Figure S2).

### 3.3 Meta-regression analyses for cycling

**FIGURE 4:**
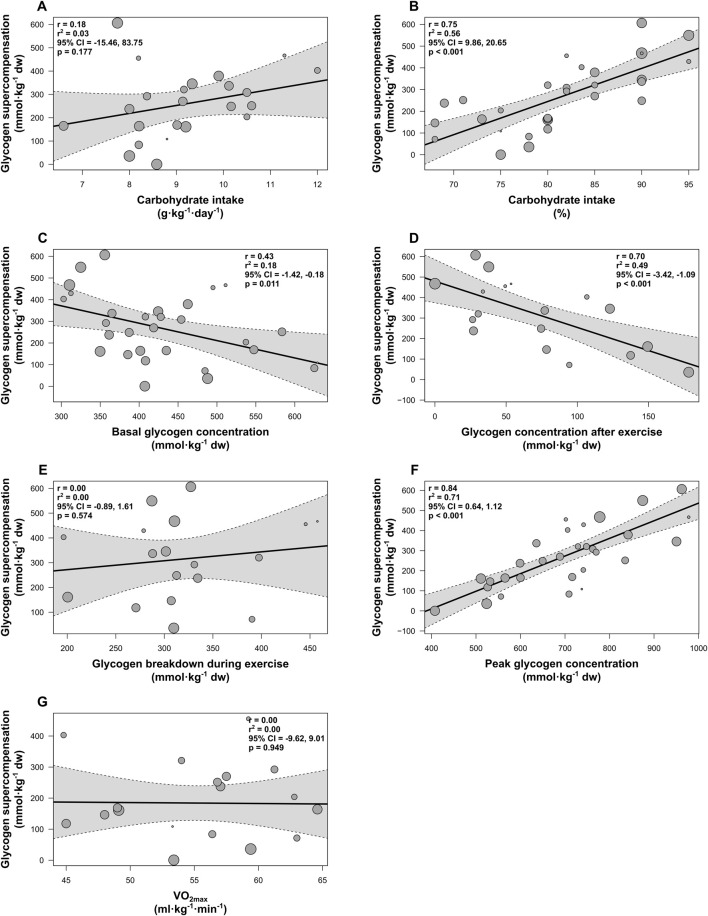
Random-effects meta-regressions showing the impact of variables on muscle glycogen supercompensation following cycling and a 3–5-day high-carbohydrate diet. **(A)** Carbohydrate intake relative to body mass (g·kg^-1^·day^-1^), **(B)** Carbohydrate intake as a percentage of total energy intake (%), **(C)** Basal glycogen concentration (mmol·kg^-1^ dw), **(D)** Glycogen concentration after exercise (mmol·kg^-1^ dw), **(E)** Glycogen breakdown during exercise (mmol·kg^-1^ dw), **(F)** Glycogen concentration during supercompensation (mmol⋅kg^-1^ dw), **(G)** VO_2max_ (ml⋅kg^-1^⋅min^-1^). Individual study groups are represented by gray circles, with circle size indicating the group’s weighting in the analysis. The solid black line represents the line of best fit, and the dotted lines denote the 95% confidence interval (CI).

Meta-regression analyses were conducted for all previously specified moderator variables. The number of included groups included in meta-regressions varied across analyses depending on the availability of data for each variable (range: 18–30 groups). Figure 4 shows the associations between these variables and muscle glycogen supercompensation following cycling and a 3–5-day high-carbohydrate diet.

A significant positive association was observed for carbohydrate intake as a percentage of total energy intake (estimate = 15.25, 95% CI [9.86, 20.65]; *p* < 0.001; R^2^ = 0.56; n = 30; Figure 4B) and for glycogen concentration during supercompensation (estimate = 0.88, 95% CI [0.64, 1.12]; *p* < 0.001; R^2^ = 0.71; n = 30; Figure 4F). Moreover, basal glycogen concentration (estimate = −0.80, 95% CI [-1.42, −0.18]; *p* = 0.011; R^2^ = 0.18; n = 30; Figure 4C) and glycogen concentration immediately after exercise (estimate = −2.25, 95% CI [-3.42, −1.09]; *p* < 0.001; R^2^ = 0.49; n = 18; Figure 4D) were significantly negatively associated with the outcome. No significant associations were found for carbohydrate intake relative to body mass (*p* = 0.177; R^2^ = 0.03; n = 23; Figure 4A), glycogen breakdown during cycling (*p* = 0.574; R^2^ = 0.00; n = 18; Figure 4E), or VO_2max_ (*p* = 0.949; R^2^ = 0.00; n = 18; Figure 4G).

#### 3.3.1 Influence of moderators on heterogeneity

A meta-regression model including carbohydrate intake as a percentage of total energy intake and glycogen concentration immediately after exercise as covariates resulted in a notable reduction in heterogeneity, with I^2^ decreasing from 92.4% to 66.1% (*p* < 0.001).

### 3.4 Publication bias and small study bias

**FIGURE 5:**
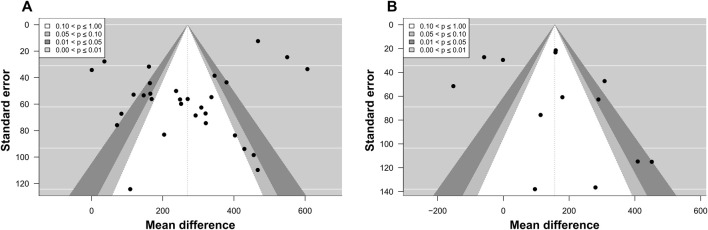
Contour-enhanced funnel plot for meta-analyses on the effects of exercise followed by a high-carbohydrate diet on muscle glycogen supercompensation. Black dots represent individual effect sizes, the vertical line represents the overall effect size, and counter-shaded areas represent p-value cutoffs, as indicated by the legend. **(A)** Funnel plot for the meta-analysis on cycling. **(B)** Funnel plot for the meta-analysis on running.

Publication bias was assessed using contour-enhanced funnel plots and Egger’s test. Visual inspection of the funnel plot revealed no apparent asymmetry. This was supported by the results of Egger’s test, which indicated no evidence of funnel plot asymmetry for cycling (*p* = 0.929; Figure 5A), and a non-significant trend toward asymmetry for running (*p* = 0.064; Figure 5B).

## 4 Discussion

This systematic review and meta-analysis examined the effects of glycogen-depleting exercise followed by a high-carbohydrate diet on skeletal muscle glycogen supercompensation. The analysis included 30 study groups investigating glycogen supercompensation after cycling, with results indicating an average increase of ∼270 mmol·kg^-1^ dw after 3–5 days on a high-carbohydrate diet. There were 13 study groups that examined glycogen supercompensation after running, showing a comparatively lower increase of ∼157 mmol·kg^-1^ dw. Meta-regression analyses for cycling revealed a positive association between the percentage of carbohydrates in the post-exercise diet and the magnitude of glycogen supercompensation. Moreover, both basal glycogen content and glycogen content immediately after exercise were negatively correlated with the magnitude of supercompensation.

We defined glycogen supercompensation as the increase in muscle glycogen content above basal levels after glycogen-depleting exercise and subsequent intake of a carbohydrate-rich diet for 3–5 days. In the 30 included study groups for cycling, comprising 207 participants, glycogen supercompensation ranged from 1 mmol·kg^-1^ dw to over 600 mmol·kg^-1^ dw, with a mean increase of 270 mmol·kg^-1^ dw. Hultman (1967) measured muscle glycogen content in 228 individuals after an overnight fast and reported that basal skeletal muscle glycogen levels varied between 237 and 640 mmol·kg^-1^ dw, with a mean of ∼358 mmol⋅kg^-1^ dw. In the studies included in this meta-analysis, the mean basal muscle glycogen content ranged from 303 to 625 mmol·kg^-1^ dw, consistent with Hultman’s findings. Our meta-regression analysis revealed a negative association between basal glycogen content and the magnitude of glycogen supercompensation.

**FIGURE 6:**
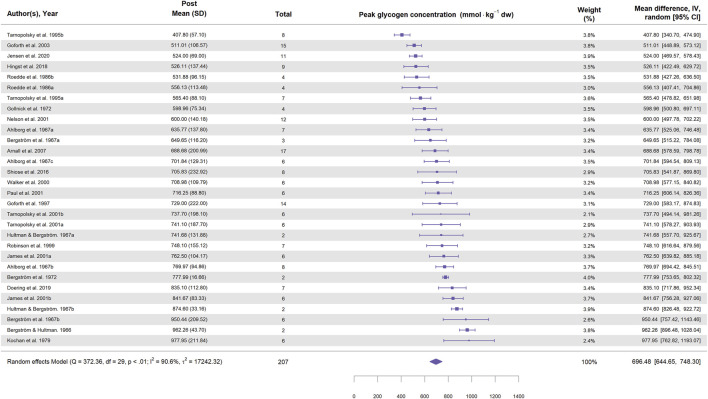
Forest plot showing glycogen concentrations during supercompensation in study groups involving cycling. Values represent glycogen concentration (mmol⋅kg^-1^ dw) with 95% confidence intervals (CI) from 22 studies (30 study groups). The diamond represents the pooled glycogen concentration (95% CI) calculated using a random-effects model. The size of each square indicates the weight of the corresponding study group in the analysis.

Glycogen content in the supercompensated state (the glycogen content reported after exercise and 3–5 days on a carbohydrate-rich diet) can also be used to describe the glycogen supercompensation. Figure 6 presents a forest plot illustrating the muscle glycogen content in the supercompensated state across the included studies for cycling. The mean glycogen content in the supercompensated state was ∼700 mmol·kg^-1^ dw, with six studies reporting values above 800 mmol·kg^-1^ dw. However, no study observed glycogen concentrations reaching 1,000 mmol·kg^-1^ dw (equivalent to 3.89 g per 100 g ww). Interestingly, the variation in glycogen content in the supercompensated state was considerably lower than the variation in glycogen supercompensation, suggesting an upper limit for glycogen storage in skeletal muscle. Supporting this notion, Hansen et al. (1999) infused high doses of glucose and insulin in two subjects, who reached a glycogen plateau of ∼1,000 mmol·kg^-1^ dw in muscle. An upper limit of muscle glycogen may also explain the negative association with basal glycogen levels, since entering the study with low glycogen will allow higher glycogen supercompensation.

Bergström and Hultman used one-legged cycling to demonstrate glycogen supercompensation and found no increase in glycogen in the rested leg (Bergström and Hultman, 1966). Therefore, the study provided evidence that glycogen supercompensation is a phenomenon restricted to previously active muscle cells, suggesting that prior glycogen depletion may be a prerequisite for supercompensation. Bergström and Hultman reported low glycogen content after exercise whereas other studies investigating glycogen supercompensation has not achieved the same low glycogen content (Roedde et al., 1986; Goforth et al., 2003; Hingst et al., 2018; Jensen et al., 2020). To examine whether the extent of glycogen depletion influences the magnitude of supercompensation, we conducted a meta-regression analysis on cycling. In the present meta-analysis, glycogen content immediately after exercise ranged from 0 to 200 mmol·kg^-1^ dw for the 18 studies that reported this. The results revealed a significant inverse correlation, indicating that lower glycogen after exercise was associated with a higher magnitude of supercompensation. These findings suggest that the extent of glycogen depletion may enhance the muscle’s ability to store glycogen beyond basal levels.

All studies reported carbohydrate intake as a percentage of total energy intake, but only 23 studies reported the absolute amount. In the studies that reported the amount of carbohydrate, the lowest intake was ∼465 g⋅day^-1^, while some reached up to ∼875 g⋅day^-1^ (∼11 g⋅kg^-1^⋅day^-1^). Meta-regression analysis showed no significant correlation between absolute carbohydrate intake per day and glycogen supercompensation, likely because all studies provided sufficient carbohydrate to maximize glycogen resynthesis. Cycling is estimated to involve ∼15 kg of muscle mass (Boushel and Saltin, 2013; McCarthy et al., 2023), and in the supercompensated state (∼4 g per 100 g wet muscle), this would correspond to an accumulation of ∼600 g of glycogen. Since the muscles were not completely depleted, all participants in the included studies may have exceeded the necessary carbohydrate intake for optimal supercompensation. To include all studies, we conducted a separate meta-regression analysis, which revealed a significant association between glycogen supercompensation and the percentage of carbohydrate in the diet. The underlying reason for this relationship remains unclear but suggests that a very carbohydrate-rich diet is recommended for glycogen supercompensation. Therefore, a carbohydrate-rich diet containing more than 8 g⋅kg^-1^⋅day^-1^ may be sufficient to cause glycogen supercompensation when no further exercise is performed.

Running and cycling were analyzed separately, because the biomechanical challenges differ during exercise. Furthermore, exhaustive running may induce less glycogen depletion and cause muscle damage (Blom et al., 1987; Madsen et al., 1990). The effect of running as a glycogen-depleting exercise prior to glycogen supercompensation with a high-carbohydrate diet has been examined in eight studies, encompassing 13 study groups and 112 participants. The mean glycogen supercompensation was ∼157 mmol·kg^-1^ dw, substantially lower than that observed after cycling. However, the magnitude of glycogen supercompensation varied greatly, ranging from a decline in glycogen levels to values comparable to those seen after cycling. Blom et al. (1987) reported lower glycogen levels after 3 days on a high-carbohydrate diet, despite low glycogen levels after exercise and a high carbohydrate intake. Notably, in this study, muscle biopsies were performed in close proximity to one another (Blom et al., 1987). Costill co-authored the paper and later published a paper demonstrating that muscle damage from biopsy procedures may impair glycogen resynthesis (Costill et al., 1988). More systematically induced muscle damage, such as that caused by eccentric exercise, has also been shown to blunt glycogen resynthesis for several days (O’Reilly et al., 1987; Costill et al., 1990; Doyle et al., 1993; Asp et al., 1995).

The present systematic review and meta-analysis is subject to several limitations that should be discussed to enhance the understanding of the results. First, despite employing a systematic search strategy across multiple databases and manually screening reference lists, the search terms used were focused on established terminology such as “supercompensation” and “carbohydrate loading”. This targeted approach may have excluded relevant studies using alternative or more recent terminology, such as “glycogen repletion”, “recovery”, or “refueling”. To mitigate this, we conducted extensive screening of reference lists from included studies. Although funnel plot analyses did not indicate the presence of publication bias, which supports the robustness of our conclusions, the potential for overlooked studies cannot be entirely ruled out.

Second, our inclusion criteria were specifically designed to focus on studies that incorporated glycogen-depleting exercise followed by a high-carbohydrate diet lasting between 3 and 5 days. Glycogen supercompensation was defined as the increase in muscle glycogen content above basal levels; therefore, studies lacking basal glycogen measurements were excluded (n = 9). While this approach ensured consistency and enabled meta-regression analyses, it also narrowed the selection of studies, possibly excluding relevant research that did not meet these criteria.

Third, the description of the high-carbohydrate diet was inadequate in some of the investigations included in this review (Hultman and Bergström, 1967; Bergström et al., 1972; Roedde et al., 1986; Robinson et al., 1999; Nelson et al., 2001; Hingst et al., 2018), with insufficient information on macronutrient quantities. This limitation restricted the inclusion of these studies in the meta-regression analysis on the role of absolute carbohydrate intake in glycogen supercompensation, thereby reducing statistical power.

Fourth, only five studies meeting the inclusion criteria have investigated glycogen supercompensation in females. We conducted a separate meta-analysis on these studies and confirmed glycogen supercompensation in females (Supplementary Figure S2; Supplementary Material). Of the three studies that compared females and males, two reported similar levels of glycogen supercompensation (James et al., 2001; Tarnopolsky et al., 2001). Tarnopolsky et al. (1995) reported supercompensation only in males, but the female participants in that study had a much lower energy intake compared to males (1974 vs. 3,364 kcal⋅day^-1^), even when adjusted for body weight. Importantly, the authors did not find gender differences when energy intake was increased (Tarnopolsky et al., 2001). The meta-analysis showed a tendency for lower glycogen supercompensation in females compared to males (p = 0.051; t-test comparing mean supercompensation in studies). Although glycogen supercompensation occurs in females, the limited representation of female participants raises questions about the generalizability of the observed magnitude, and the results should be interpreted with caution. Future studies with balanced gender representation of should be conducted to clarify the question.

Finally, the I^2^ statistic was greater than 75% for the analyses of the effects of both cycling and running exercises on muscle glycogen supercompensation. This high level of heterogeneity indicates substantial variability among the included studies, likely due to differences in exercise and nutritional protocols. This assumption is partially supported by the meta-regression model for cycling that included percentage carbohydrate intake and glycogen immediately after exercise as moderator variables. Despite the high heterogeneity, most of the included studies reported glycogen supercompensation, lending credibility to the overall conclusions of this review. However, this variability also limits the generalizability of the findings, as the observed effects may differ depending on variables such as basal glycogen content, exercise protocol, glycogen depletion, and dietary strategies. The high I^2^ values reduce the precision of the pooled estimates, and these findings should therefore be interpreted with caution. Future studies should aim to standardize protocols to reduce heterogeneity and improve comparability across research.

Future research should investigate the mechanisms that limit glycogen storage in skeletal muscle. Animal studies have shown that overexpression of GLUT1 or an activated glycogen synthase causes glycogen supercompensation (Ren et al., 1993; Manchester et al., 1996), suggesting particularly focusing on glucose uptake and glycogen synthase activity. Hingst et al. (2018) investigated insulin-stimulated glucose uptake during glycogen supercompensation, but did not find higher insulin-stimulated glucose uptake in the exercise leg. Since the glycogen supercompensation occurs over 3–5 days, a large increase in glucose uptake is not necessary to increases glycogen storage. Instead, it may be more likely that glucose directions to glycogen synthesis in the reason for glycogen supercompensation. It has been demonstrated that muscle contraction activates glycogen synthase independently of the decline in glycogen content which could potentially stimulate glycogen supercompensation (Lai et al., 2007). Among the studies included in this review, three measured glycogen synthase activity, and all reported increased activity immediately after exercise when glycogen was low (Bergström et al., 1972; Kochan et al., 1979; Hingst et al., 2018). The idea that this elevated glycogen synthase activity may contribute to glycogen supercompensation is further supported by Hingst et al. (2018), who reported elevated glycogen synthase activation the day after exercise when glycogen stores were repleted.

Hingst et al. (2018) reported expression data on more than 3,300 proteins during glycogen supercompensation and found upregulation of GLUT1, GLUT4, and hexokinase II, supporting that elevated glucose uptake could contribute to supercompensation. Many questions about glycogen remain unanswered (Jensen and Kolnes, 2024) and future research should characterize the time-course for protein phosphorylation and transcription with new omics’ technologies. Phosphoproteomics has been used successfully on skeletal muscles after exercise and reported phosphorylation of numerous proteins (Hoffman et al., 2015; Needham et al., 2022), and might help clarify potential signaling mechanisms. Time-course studies of global RNA sequencing would allow to detect transcription patterns and maybe identify new candidate proteins involved in supercompensation. The impact of such studies might go beyond glycogen supercompensation, because glycogen can be accurately quantified and related to patterns of phosphorylation and transcription. Electron microscopical analyses have shown that glycogen particles in skeletal muscle are localized in three pools: intermyofibrillar, intramyofibrillar, and subsarcolemmal (Jensen et al., 2022). Future research should characterize size and localization of the glycogen particles in glycogen supercompensated muscles.

The significant glycogen supercompensation from basal levels after cycling or running paired with a high-carbohydrate diet has important implications for sports nutrition and training practices. These findings underscore the effectiveness of tailored high-carbohydrate diets in enhancing muscle glycogen supercompensation, especially following glycogen-depleting activities. Coaches and athletes should integrate specific carbohydrate intake strategies based on the type of exercise performed, as cycling appears to result in greater glycogen storage compared to running.

## 5 Conclusion

In conclusion, muscle glycogen supercompensation occurs following both cycling and running after 3–5 days on a high-carbohydrate diet, with a greater magnitude observed after cycling compared to running. The magnitude of glycogen supercompensation after cycling is influenced by basal glycogen levels, glycogen content after exercise, and the relative carbohydrate content of the diet.

## Data Availability

The original contributions presented in the study are included in the article/Supplementary Material, further inquiries can be directed to the corresponding author.

## Supplementary material

SUPPLEMENTARY TABLE S1
Search strings used for literature retrieval in PubMed and Web of Science. The table displays the complete search strategies applied in PubMed and Web of Science to identify relevant studies on muscle glycogen supercompensation in humans. The search was conducted without any restriction for the year of publication, and the language filter was used to select only studies written in English.

SUPPLEMENTARY FIGURE S1
Forest plot showing the effect of cycling followed by a 3–5-day high-carbohydrate diet on muscle glycogen supercompensation in males. Effect sizes are expressed as mean difference (MD) in glycogen concentration (mmol⋅kg^-1^ dw) with 95% confidence intervals (CI) from 19 studies (24 study groups). The diamond represents the pooled effect size (95% CI) calculated using a random-effects model. The size of each square indicates the weight of the corresponding study group in the analysis.

SUPPLEMENTARY FIGURE S2
Forest plot showing the effect of cycling followed by a 3–5-day high-carbohydrate diet on muscle glycogen supercompensation in females. Effect sizes are expressed as mean difference (MD) in glycogen concentration (mmol⋅kg^-1^ dw) with 95% confidence intervals (CI) from 5 studies (5 study groups). The diamond represents the pooled effect size (95% CI) calculated using a random-effects model. The size of each square indicates the weight of the corresponding study group in the analysis.
