# Supplementary material for: Glycogen supercompensation in skeletal muscle after cycling or running followed by a high carbohydrate intake the following days: a systematic review and meta-analysis
Source: Front Physiol. 2025 Aug 18;16:1620943. doi: 10.3389/fphys.2025.1620943 (PMC12399638; doi:10.3389/fphys.2025.1620943)
Supplement: Supplementary file 2 [file Table1.docx]

**Table S1. Search strings used for literature retrieval in PubMed and Web of Science.** The table displays the complete search strategies applied in PubMed and Web of Science to identify relevant studies on muscle glycogen supercompensation in humans. The search was conducted without any restriction for the year of publication, and the language filter was used to select only studies written in English.

| **Database** | **Search String** |
| --- | --- |
| PubMed | (("muscle s"[All Fields] OR "muscles"[MeSH Terms] OR "muscles"[All Fields] OR "muscle"[All Fields]) AND ("glycogen"[Supplementary Concept] OR "glycogen"[All Fields] OR "glycogen"[MeSH Terms] OR "glycogenic"[All Fields] OR "glycogens"[All Fields]) AND ("supercompensated"[All Fields] OR "supercompensation"[All Fields] OR "Hultman"[All Fields] OR ("diet, carbohydrate loading"[MeSH Terms] OR ("diet"[All Fields] AND "carbohydrate"[All Fields] AND "loading"[All Fields]) OR "carbohydrate loading diet"[All Fields] OR ("carbohydrate"[All Fields] AND "loading"[All Fields]) OR "carbohydrate loading"[All Fields])) AND ("human s"[All Fields] OR "humans"[MeSH Terms] OR "humans"[All Fields] OR "human"[All Fields])) NOT ("brain"[MeSH Terms] OR "brain"[All Fields] OR "brains"[All Fields] OR "brain s"[All Fields] OR ("liver"[MeSH Terms] OR "liver"[All Fields] OR "livers"[All Fields] OR "liver s"[All Fields])) |
| Web of Science | ((ALL=(Muscle glycogen) AND (ALL=(supercompensation) OR AI=(Hultman) OR ALL=(carbohydrate loading))) AND ALL=(human)) NOT ALL=(brain) NOT ALL=(liver) |
