# Supplementary figures and images for: Glycogen supercompensation in skeletal muscle after cycling or running followed by a high carbohydrate intake the following days: a systematic review and meta-analysis
Source: Front Physiol. 2025 Aug 18;16:1620943. doi: 10.3389/fphys.2025.1620943 (PMC12399638; doi:10.3389/fphys.2025.1620943)

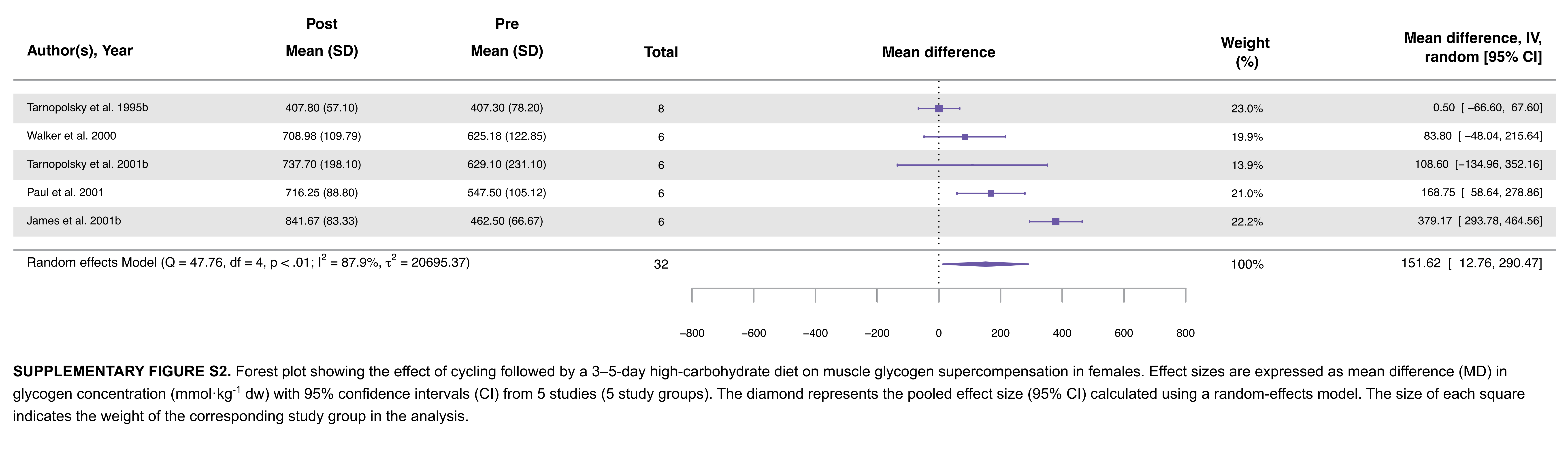

Supplement: Supplementary file 3 [file Image2.tiff]
